# Response to crizotinib treatment for ROS1 p.H1999N mutation and secondary EGFR p.V774M mutation after drug resistance: a Case Report

**DOI:** 10.3389/fphar.2026.1777570

**Published:** 2026-05-08

**Authors:** Yalin He, Yuan Tang, Ruixuan Yu, Yongmei Liu

**Affiliations:** 1 Division of Thoracic Tumor Multimodality Treatment, Cancer Center, West China Hospital, Sichuan University, Chengdu, China; 2 Department of Pathology, West China Hospital, Sichuan University, Chengdu, Sichuan, China

**Keywords:** EGFR p.V774M mutation, epidermal growth factor receptor, lung adenocarcinoma, next-generation sequencing, ROS1 p.H1999N mutation

## Abstract

Genetic fusion involving the ROS1 gene lead to the constitutive activation of its encoded tyrosine kinase, thereby acting as an oncogenic driver that promotes tumor cell proliferation. Numerous missense mutations in ROS1 are currently classified as variants of unknown significance, especially, mutations in kinase domain are crucial mediators of the resistance of ROS1 fusion-positive cancers to ROS1 tyrosine kinase inhibitors (TKIs). This study presents a rare case of a ROS1 p.H1999N mutation positive lung adenocarcinoma in a male patient, who has benefited from crizotinib treatment. Following 9 months of crizotinib treatment, chest computed tomography (CT) revealed disease progression. The pathological results of the second tissue biopsy indicated ROS1 p.H1999N mutation accompanied by EGFR p.V774M mutation. Subsequently, the patient received furmonertinib targeted therapy. This study highlights the importance of comprehensive genetic sequencing for clinical decision-making and underscores crizotinib’ potential as the first-line target-therapy for ROS1 missense mutations tumors. Herein, we discuss the diagnostic challenges and the potential pathogenic mechanisms of this novel mutation. How to identify rare genes and translate their identification into clinical benefits is a worthwhile avenue for exploration in our future work.

## Introduction

Approximately 1%–2% of NSCLCs harbor ROS1 fusion, and these patients are more frequent in females, younger, and nonsmokers with a histologic diagnosis of adenocarcinoma (Bergethon et al., 2012; Marchetti et al., 2017). Patients with ROS1 fusion in advanced lung cancer have received clinically significant benefits from the emergence of ROS1-TKI. The objective response rate (ORR) was 72% [95% confidence interval (CI), 58%–83%], and median progression-free survival was 19.3months (95% CI, 15.2–39.1) based on the updated PROFILE 1001 analysis (Shaw et al., 2019). However, point mutations in ROS1 are often accompanied by gene fusion and are considered the cause of drug resistance. Because it is extremely rare, the biological effects of simple ROS1 point mutations are not clear. One of the inevitable challenges during targeted therapy is the phenomenon of secondary resistance. Repeated tissue biopsy is important to explore resistance mechanisms.

Herein, we present the first case of a ROS1 p.H1999N mutation occurring without ROS1 fusion in lung adenocarcinoma and the clinical efficacy of crizotinib therapy. After developing resistance to crizotinib, the patient emerged co-mutation of ROS1 p.H1999N and EGFR p.V774M. The patient succumbed to tracheoesophageal fistula complicated with severe pulmonary infection 1 week following the initiation of EGFR-TKI targeted therapy.

## Case presentation

**FIGURE 1:**
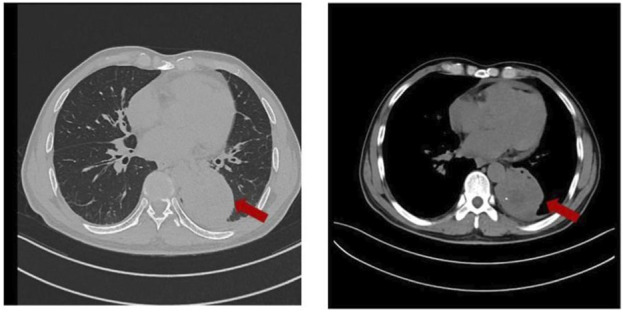
Chest CT scans showed the primary lesions (red arrowheads) in the lung on 24 December 2024.

**FIGURE 2:**
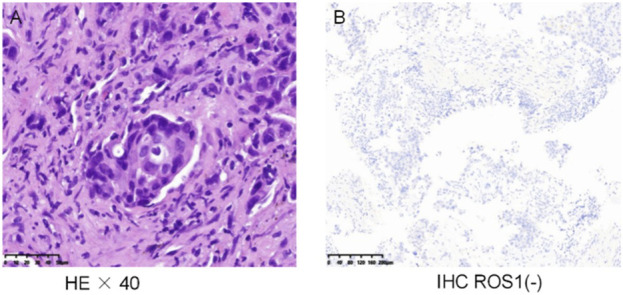
Histological, immunohistological findings of the primary lung. **(A)** The biopsy specimen showed adenocarcinoma lung cancer. **(B)** No expression of ROS1 (staining not shown).

A 59-year-old Chinese man was admitted to a Tertiary A Hospital on 20 December 2024, due to a 1-month history of cough and a 1-week history of hemoptysis. The patient has a 30-year history of smoking and had not quit smoking at the time of visiting the hospital. He denied other prior disease and cancer family history. The CT scan showed a soft tissue mass in the lower lobe of the left lung, approximately 7.3 * 5.8 cm in size, accompanied by obstructive atelectasis, obstructive pneumonia, and left pleural effusion (Figure 1). Lymph node metastasis is present in bilateral hilum, mediastinum, and bilateral supraclavicular regions. Cranial magnetic resonance imaging (MRI)showed metastatic lesions in the left parietal lobe, the right parietal lobe, and the cerebral falx. Abdominal CT scan and 99mTc-Methylene Diphosphate (MDP) bone scintigraphy images did not reveal any signs of tumors. A painless fiberoptic bronchoscopy was performed, and the biopsy pathologic diagnosis was lung adenocarcinoma (Figure 2A). Immunohistochemistry findings indicated CK(+),CK7(+),TTF-1 (+),Napsin-A (+),CD56 (−),P63 (−),Ki-67 (approximately 70%+),ALK (D5F3) (−) and ROS1(−) (Figure 2B). With written informed consent, the formalin fixed paraffin embedded (FFPE) tissues in the biopsy specimen were detected by targeted DNA-based NGS of 56-gene panel. (Burning Rock Biotech, Guangzhou, People’s Republic of China). The mutation profile showed a ROS1 p.H1999N mutation (allelic frequency of 26.55%) and a TP53 p.C275Y mutation (allelic frequency of 46.82%). The final diagnosis was stage IV left lung adenocarcinoma (cT4N3M1).

**FIGURE 3:**
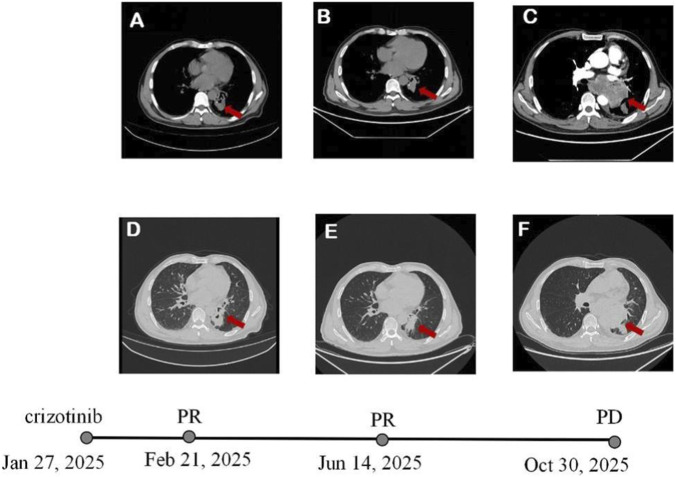
Variations of the primary lesion in lung detected by chest CT scans during treatment (red arrowheads). **(A,D)** One month after treatment with crizotinib. **(B,E)** Four months after treatment with crizotinib. **(C,F)** Nine months after treatment with crizotinib.

The patient was treated with the gamma knife to brain lesions on 3 January 2025, and crizotinib medication (250 mg, twice daily, oral) from 27 January 2025. After a month, a follow-up chest CT scan showed a notable decrease in tumor size of 4.6 * 3.7 cm. The lymph nodes shrank significantly, and the pleural effusion disappeared (Figures 3A,D). The patient achieved a partial response according to the Response Evaluation Criteria In Solid Tumors v1.1. Subsequent evaluations revealed tumor stability (Figures 3B,E). After 9 months of progression-free survival period, a follow-up chest CT showed progression of the pulmonary (Figures 3C,F). A bronchoscopy biopsy was conducted again, and the pathological findings indicated non-small-cell lung carcinoma. From that time on, the patient stopped taking crizotinib and underwent one cycle of pemetrexed and cisplatin (AC) chemotherapy. After that, the results of genetic testing indicated ROS1 p.H1999N mutation (allelic frequency of 28.18%), EGFR p.V774M mutation (allelic frequency of 11.19%) and TP53 p.C275Y mutation (allelic frequency of 29.86%). After the first cycle of chemotherapy, the patient developed a tracheoesophageal fistula and the side effects were unbearable. The patient chose oral treatment with furmonertinib at 80 mg QD on 11 December 2025. Unfortunately, 1 week after the targeted treatment, the patient died due to an uncontrollable esophageal-tracheal fistula and pulmonary infection.

## Discussion

**FIGURE 4:**
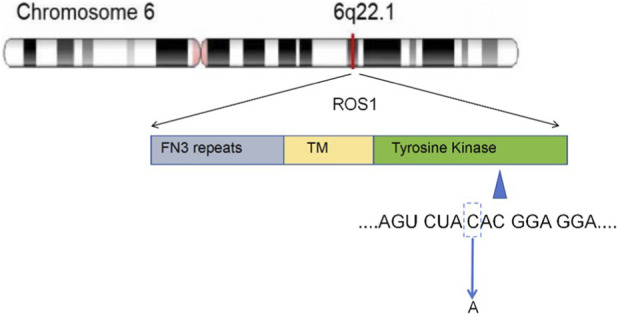
The SNV diagram reveals a point mutation in the tyrosine activation domain of the ROS1 gene, where the cytosine (C) at position 5595 is substituted with adenine (A).

ROS1 consists of an extracellular N-terminal domain, a transmembrane domain, and a C-terminal intracellular tyrosine kinase structural domain (Yu et al., 2022). At least 55 different 5′ gene partners have been identified in fusion with the 3′ regions of ROS1 (Drilon et al., 2021). The complete retention of the kinase domain is a common feature of various ROS1 fusion proteins. Even though point mutations are described in many cancer entities, like EGFR p.L858R, point mutations of ROS1 are thought to be the mechanism that leads to secondary resistance to crizotinib (McCoach et al., 2018). An aspartic acid-to-asparagine substitution occurs at ROS1 codon 2033 (p.D2033N) within the ATP-binding site, leading to the abrogation of crizotinib binding through a powerful modification of the electrostatic forces in the ATP-binding site (Drilon et al., 2016). Similar to the ALK p.G1202R mutation of non-small cell lung cancer, the ROS1 p.G2032R mutation, located in the solvent front of the kinase domain, is the strongest (Awad et al., 2013). It allows continuous ATP binding, but causes spatial conflict with the piperidine ring of crizotinib (Gou et al., 2018). p.S1986F/Y, p.L2026M, and p.L2155S can also lead to drug resistance through different pathways. The ROS1 p.H1999N mutation refers to the amino acid at position 1999 of the ROS1 protein changing from histidine (His) to asparagine (Asn). This variation is located in the protein kinase domain of the ROS1 protein (Figure 4). An *in vivo* experiment and a cell-based *in vitro* model confirmed that ROS1 p.D2113N which aspartic acid (Asp)is replaced by asparagine (Asn) at position 2113 in the ROS1 kinase domain, can be an oncogenic variant and sensitive to ROS1-TKI. The carcinogenic mechanism may be that the ROS1 p.D2113N mutation dramatically enhances the flexibility of the A-loop, thereby increasing the proportion of the active conformation of the kinase (DFG-in) (Iyer et al., 2023). This trial provided preclinical data on the correlation between only ROS1 point mutations and malignant tumors that are responsive to tyrosine kinase inhibitors. Interestingly, Lisa Velthaus et al. reported a case of a pancreatic cancer patient with a novel ROS1 p.L1950F mutation without ROS1 rearrangement, who benefited from ROS1-TKI with a PFS of 12 months (Velthaus et al., 2021). That case further verified that the point mutation in the ROS1 tyrosine kinase domain might be an oncogenic driver in pancreatic carcinoma. However, the limitation is that NGS was not performed to rule out the existence of ROS1 fusion in this case. According to the cBioPortal database, pancreatic cancer patients with ROS1 point mutations are all accompanied by KRAS mutations. In cases with or without co-mutations of other oncogenes, the carcinogenic ability of ROS1 still needs to be further explored.

Common EGFR mutations primarily consist of exon 19 deletions and the p.L858R mutation. Other recurrent alterations include exon 20 insertions and the p.T790M mutation, the latter of which often emerges as a resistance mechanism to TKIs (Tavernari et al., 2025). Other uncommon mutations (occurs at the P-loop (L718–V726) and the C-terminal loop of the αC-helix (A767–G779)) represent approximately 10%–15% of EGFR mutations and generally exhibit a lower response to TKIs (Robichaux et al., 2021; Borgeaud et al., 2024). EGFR p.V774M mutation was characterized as P-loop and αC-helix compressing (PACC) mutation. The clinical significance of the p.V774M mutation remains unclear. Previous case reports have confirmed that patients with the p.V774M mutation, either alone or in combination with other oncogenic mutations, benefited from osimertinib targeted therapy (Zhuang et al., 2023; Cosi et al., 2024; Yang et al., 2018). Therefore, the third generation of EGFR-TKIs may be a treatment option to p.V774M mutant NSCLC. In this case, owing to the extremely short duration of EGFR-TKI administration, we were unable to evaluate whether the patient achieved a clinical response to the treatment.

TP53 is the most common co-mutation type in NSCLC, with an incidence rate of approximately 48% (Li et al., 2023). The presence of TP53 mutations is related to tumor progression and metastasis, which translates to shorter overall survival (Van E et al., 2022). For patients with ROS1-positive non-small cell lung cancer who received crizotinib as the first-line treatment, compared with wild-type patients, TP53 co-mutant patients had a statistically significantly shorter median PFS (7.0 months vs. 24.1 months) (Michels et al., 2019). Among patients receiving EGFR-TKI or ALK-TKI targeted therapies, those with TP53 co-mutations also showed poorer survival rates (Wei et al., 2025; Qin et al., 2020). In this patient, despite the favorable response to first-line targeted therapy, the presence of both TP53 mutations and rare mutations likely contributed to their relatively short overall survival.

NGS can fully sequence all types of mutations in a large number of genes (hundreds to thousands), thus improving the detection rate of rare genes (Sabour et al., 2017). In this case, although ROS1 IHC is negative, DNA-based NGS showed positive results. Considering the heterogeneity and the superiority of RNA-based NGS in fusion detection (Pavlakis et al., 2019; Li et al., 2025). Further RNA-based NGS was performed on the primary tumor tissue, which showed no ROS1 fusion, thus confirming the presence of a ROS1 missense mutation rather than a fusion event. DNA-based NGS focuses on coding sequences, while RNA-based NGS surveys exons (Lindeman et al., 2018). Therefore, their results are not contradictory but complementary. Owen et al. demonstrated that combined DNA- and RNA-based NGS increased the detection rate of actionable structural variants by 15.3% compared with DNA-based NGS alone (Owen et al., 2024). The combination of DNA and RNA-based NGS is expected to discover novel variations of unknown significance in confirmed driver genes (such as ROS1). Functional mutations and passenger mutations were further distinguished through clinical practice. Taken together, the wide use of NGS has expanded the map of drug-sensitive genetic variations and also increased the proportion of people benefiting from targeted treatment.

In conclusion, we report a case of metastatic lung adenocarcinoma characterized by the ROS1 p.H1999N mutation, which has conventionally been considered a non-driver mutation, and demonstrate its partial response to ROS1-TKI. The far undescribed missense mutation was determined by DNA-based NGS of 56 targeted genes, and ROS1 fusion was excluded by further RNA-based NGS. After 9 months of crizotinib treatment, drug resistance developed. DNA-based NGS revealed an additional EGFR p.V774M mutation. Given the rapid progression of the disease, evaluation of the patient’s response to EGFR-TKI treatment was not feasible. This report provided the clinical evidence on this newfound oncogenic ROS1 missense mutation and offered drug-sensitive support for targeted therapy in lung adenocarcinoma. It also emphasizes the importance of genetic testing in clinical applications to maximize the advantages of targeted therapy.

## Data Availability

The raw data supporting the conclusions of this article will be made available by the authors, without undue reservation.
